# Predictive value of triglycerides to high-density lipoprotein cholesterol and triglyceride glycemic index for diabetes incidence in pre-diabetes patients: a prospective cohort study

**DOI:** 10.1186/s41043-023-00410-5

**Published:** 2023-07-11

**Authors:** Mehdi Sharafi, Zahra Amiri, Babak Pezeshki, Mohammad Ali Mohsenpour, Mohammad Hassan Eftekhari, Sima Afrashteh, Elham Haghjoo, Akram Farhadi, Mohsen Khaleghi, Zahra Mastaneh

**Affiliations:** 1 Social Determinants in Health Promotion Research Center, Health Institute, Hormozgan University of Medical Sciences, Bandar Abbas, Iran; 2 Student Research Committee, Shiraz University of Medical Sciences, Shiraz, Iran; 3 Department of Clinical Nutrition, School of Nutrition and Food Sciences, Shiraz University of Medical Sciences, Shiraz, Iran; 4 Department of Public Health, School of Public Health, Bushehr University of Medical Sciences, Bushehr, Iran; 5 Department of Persian Medicine, Fasa University of Medical Sciences, Fasa, Iran; 6 The Persian Gulf Tropical Medicine Research Center, The Persian Gulf Biomedical Sciences Research Institute, Bushehr University of Medical Sciences, Bushehr, Iran; 7 Department of Mathematics, Fasa Branch, Islamic Azad University, Fasa, Iran; 8 Health Information Management, School of Allied Medical Sciences, Hormozgan University of Medical Sciences, Bandar Abbas, Iran

**Keywords:** Triglyceride glucose index, Triglyceride-to-high-density lipoprotein cholesterol ratio, Cox regression model, Pre-diabetes, Diabetes, Fasa Persian cohort study

## Abstract

**Background:**

The triglyceride glucose (TyG) and triglyceride-to-high-density lipoprotein cholesterol ratio (TG/HDL-c) are the indices that can predict the progression of pre-diabetes to type 2 diabetes mellitus (T2DM). This study aimed to examine the relationship between TyG and TG/HDL-c indices with the incidence of T2DM in pre-diabetes patients.

**Methods:**

A total of 758 pre-diabetic patients aged 35–70 years who were enrolled in a prospective Fasa Persian Adult Cohort were followed up for 60 months. TyG and TG/HDL-C indices were obtained at baseline data and divided into quartiles. The 5-year cumulative incidence of T2DM was analyzed by Cox proportional hazards regression analysis while controlling for baseline covariates.

**Results:**

During 5 years of follow-up, there were 95 incident cases of T2DM, with an overall incidence rate of 12.53%. After adjusting for age, sex, smoking, marital status, socioeconomic status, body mass index, waist circumference, hip circumference, hypertension, total cholesterol, and dyslipidemia, the multivariate-adjusted hazard ratios (HRs) demonstrated that patients with the highest TyG and TG/HDL-C indices quartile were at higher risk of T2DM (HR = 4.42, 95%CI 1.75–11.21) and (HR = 2.15, 95%CI 1.04–4.47), respectively, compared to participants in the lowest quartile. As the quantiles of these indices increase, the HR value shows a significant increment (*P* < 0.05).

**Conclusion:**

The results of our study showed that the TyG and TG/HDL-C indices can be important independent predictors for the progression of pre-diabetes to T2DM. Therefore, controlling the components of these indicators in pre-diabetes patients can prevent developing T2DM or delay its occurrence.

## Introduction

Type 2 diabetes mellitus (T2DM) is a serious health concern for health systems and health providers around the world due to its high prevalence, rapid growth, and life-threatening complications [1]. According to the latest diabetes atlas by the International Diabetes Federation (IDF), 10.5% of adults aged 20–79 years were suffered from diabetes in 2021 globally, and it is expected to rise to 12.2% by 2045 [2]. Reports in 2019 stated that T2DM was the direct cause of death for 1.5 million individuals, and 48% of all diabetes-related deaths occurred before age 70 years [3]. A large number of diabetes-related deaths are caused by macro-vascular complications including stroke and heart disease [4]. Therefore, providing a screening model and strategies is necessary for prevention, diagnosis, and medical treatment in the early stages [5].

Given that insulin resistance is the main pathological mechanism of T2DM [6], alternative indices of insulin resistance such as triglyceride glucose (TyG) index and triglyceride-to-high-density lipoprotein cholesterol ratio (TG/HDL-c) can be helpful in the prediction of T2DM development [7, 8]. The results of several studies, including studies conducted on American, European, and Asian populations, showed that although fat deposition is affected by race and ethnicity, these indicators are significantly related to insulin resistance [9–13]. TyG is observed to reflect insulin resistance and identify individuals with a high risk of developing cardiovascular diseases [14]. TyG index is calculated using the measurement of fasting triglyceride (TG) and fasting blood glucose (FBG) (Ln (TG ∗ FBS)/2) [15]. The TyG index is proposed as a simple and low-cost indicator of insulin resistance [15]. It was seen individuals in higher TyG quartiles are 3.67 times higher in the risk of developing T2DM [5]. Moreover, it was shown that the risk of T2DM for the highest quartile of the TG/HDL-C, after adjustment of confounding variables, is 2.54 times higher in comparison with the lowest quartile [16]. Also, another study indicated individuals in the highest quartile of the TyG index, after adjusting for confounding variables, have a 3.67 higher risk of T2DM development in comparison with the lowest quartile [5].

Despite the observed relationship between TyG and TG/HDL-C indices and diabetes incidence, few studies in Iran have investigated the above-mentioned association. To the best of our knowledge, considering the high incidence rate for T2DM [17], these indices have not been studied in the south region of Iran. Also, the spectrum of TyG and insulin resistance levels vary according to ethnicity [18]. Therefore, the present study aimed to determine the relationship between TyG and TG/HDL-C indices with the risk of T2DM in pre-diabetic patients.

## Materials and methods

### Study population

The present prospective cohort study was carried out on the baseline data of the Fasa Persian Adult Cohort Study (FACS) and a 60-month follow-up of participants. Of FACS participants, 35–70-year-old pre-diabetic patients were recognized and their data were extracted from the FACS database. The FACS is a branch of the Persian Cohort Study, which was initiated in 2013 to investigate the risk factors for cardiovascular diseases in the Sheshdeh and Qara-Balagh regions containing 22 villages, Fasa, Fars province, Iran.

Briefly, 10,000 adults aged 35–70 years who were residing in the above-mentioned area formed the initial sample size of the FACS. Phone calls and referrals from health centers located in the region were used to invite individuals to the study center. To record baseline data, an average of 20 people were presented in the center.

A comprehensive questionnaire was designed for data gathering. The questionnaire was filled by the assessments and interviews done by trained personnel. Using the pre-designed questionnaire, FACS’s personnel recorded demographic information, socioeconomic status (SES), medical and clinical information, history of communicable and non-communicable diseases, and nutritional intakes. A separate unique interviewer was considered to complete each part of the questionnaire based on the personnel’s area of expertise. Also, blood samples have been collected, stored, and analyzed. Collected data and blood sample analysis results are stored in an electronically central data server daily. The baseline data collection was completed in 2016, and six follow-up periods have been done so far. Further details of the FACS are available at the FASA cohort study protocol [19].

### Variables of the study

#### Pre-diabetes and T2DM

Participants in the FASA Persian cohort study, whose baseline fasting blood sugar (FBS) was between 100 and 125 mg/dl, were known as pre-diabetic. The pre-diabetic participants were followed up for 60 months for the incidence of T2DM. The incidence of T2DM is confirmed in pre-diabetic individuals by an annual assessment of the fasting blood sugar using the criteria developed by the Diabetes Association Center. Therefore, FBS over 125 mg/dl was considered borderline for diabetes [20].

#### TyG and TG/HDL-c

Fasting blood sugar (FBS) and triglycerides (TG) were used to calculate the TyG index based on its standard formula: ln[TG (mg/dL) × FBG (mg/dL)/2]. Moreover, TG was divided by high-density lipoprotein (HDL) to calculate TG/HDL-c index [21].

#### Demographic and socioeconomic status (SES)

Demographic characteristics of participants including gender, age, marital status, and education level were extracted from the FACS’s database. To calculate SES, the principal component analysis (PCA) method based on the lifestyle and household-related variables including the ownership of a house, freezer, washing machine, dishwasher, personal computer (PC), car, motorcycle, color TV, vacuum cleaner, and cell phone, house area, number of rooms in the house, bathroom, family size, Internet access, car price, using a laptop, PC, Internet, car, number of books read, foreign and domestic pilgrim, and foreign and domestic travels were used. According to the results of PCA, individuals were categorized into low, middle, and high SES [22, 23].

#### Clinical and medical history

Weight, height, body mass index (BMI), waist circumference (WC), hip circumference (HC), lipid profile (including low-density lipoprotein (LDL), HDL, and TG), and cardiovascular disorders including dyslipidemia (dyslipidemia was defined as LDL ≥ 130 mg/dL, or TC ≥ 200 mg/dL, or HDL ≤ 40 mg/dL in men, and 50 mg/dl in women or TG ≥ 150 mg/dL and or use of lipid-lowering medications in the past two weeks) [24], and hypertension (defined as systolic BP ≥ 140 mmHg and/or diastolic BP ≥ 90 mmHg) [25] were obtained from the data center. Moreover, the 5-year incidence rate of T2DM in pre-diabetic individuals was calculated in regard to its quartiles and their 95% confidence intervals (CI).

### Statistical analysis

For quantitative variables, the normality of the data was checked by the Shapiro test (*p* > 0.05) and Q–Q plot. To report the study variables in the TyG and TG/HDL-c quartile, mean and standard deviation (mean ± SD), and frequency and percentage were used for quantitative variables and qualitative variables, respectively. To investigate the differences between the TyG and TG/HDL-c quartile, one-way ANOVA and Chi-square tests were used. The median survival time (T2DM incidence) was calculated using the Kaplan–Meier chart, and the log-rank test was performed to compare the median survival time in the TyG and TG/HDL-c index quartiles.

The Cox proportional hazard model was used as the most common model in survival studies to evaluate the relationship between TyG and TG/HDL-c indices with the incidence of T2DM during 60 months or 5 years of the follow-up period. To control the effect of confounders in this study, other covariates (age, gender, smoking, marital status, SES, BMI, WC, HC, hypertension, total cholesterol, dyslipidemia, HDL) were included in the model. The adjusted hazard ratio with its 95% confidence interval was used to report the association. In all analyses, the first type error (α) was considered 0.05. A *p*-value lower than 0.05 is considered significant.

## Results

**Fig. 1:**
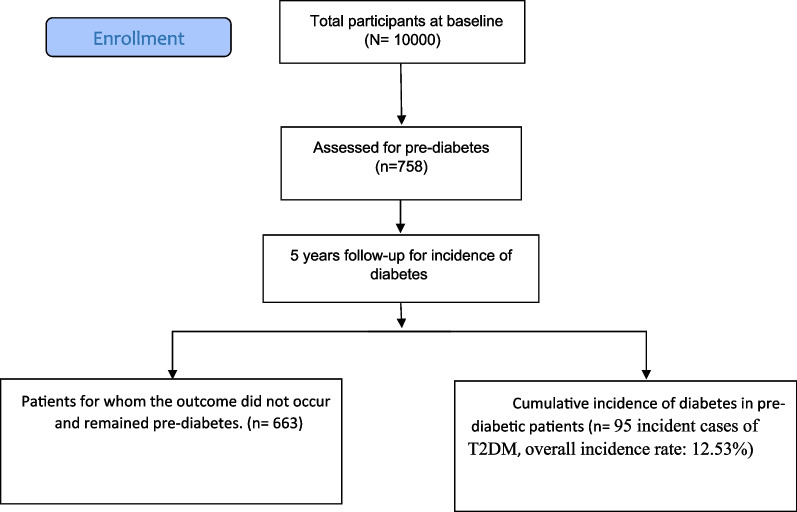
Flow diagram of study population

In the present study, a total of 758 participants (59.23% were female), aged 52.57 ± 9.22 years (mean ± SD), with pre-diabetes at baseline assessment of FACS were enrolled. The flow diagram of the study population is shown in Fig. 1.

The baseline characteristics of participants including anthropometric measurements, laboratory examinations, and demographic characteristics according to the quartile of TyG and TG/HDL-c indices are shown in Tables 1 and 2.

**Table 1:** Baseline characteristics of participants, based on the TyG index quartiles in pre-diabetes patients aged 35–70 years

Variables	TyG index quartiles				*P*-value
	Q1	Q2	Q3	Q4	
*N*	190	189	190	189	
Age (years)	51.61 ± 10.11	53.30 ± 8.67	53.81 ± 8.52	51.56 ± 9.35	0.030
Sex					
Men	75 (39.5)	71 (37.6)	75 (39.5)	88 (46.6)	0.297
Women	115 (60.5)	118 (62.4)	115 (60.5)	101 (53.4)	
Marital status					
Single	35 (18.4)	23 (12.2)	26 (13.7)	29 (15.3)	0.359
Married	155 (81.6)	166 (87.8)	164 (86.3)	160 (84.7)	
Education (year)	4.10 ± 3.90	3.25 ± 3.59	3.94 ± 3.79	3.78 ± 3.74	0.145
Socioeconomic status					
Low	84 (44.2)	69 (36.5)	73 (38.4)	84 (44.4)	0.571
Moderate	34 (17.9)	35 (18.5)	37 (19.5)	37 (19.6)	
High	72 (37.9)	85 (45.0)	80 (42.1)	68 (36.0)	
Body mass index (kg/m2)	24.96 ± 4.91	27.18 ± 5.48	27.85 ± 4.79	28.13 ± 4.66	< 0.001
Waist circumference (cm)	91.31 ± 12.31	97.41 ± 12.94	99.08 ± 11.84	100.13 ± 12.43	< 0.001
Hip circumference(cm)	98.37 ± 9.61	101.73 ± 10.29	102.00 ± 9.19	102.51 ± 9.05	< 0.001
Smoking					
Yes	41 (21.6)	45 (23.8)	42 (22.1)	45 (23.8)	0.934
No	149 (78.4)	144 (76.2)	148 (77.9)	144 (76.2)	
Glucose (mg/dL)	106.90 ± 8.61	109.88 ± 11.72	116.39 ± 26.22	128.76 ± 46.75	< 0.001
Total cholesterol (mg/dL)	177.07 ± 36.16	191.85 ± 36.76	206.20 ± 38.45	218.07 ± 50.45	< 0.001
HDL cholesterol (mg/dL)	58.88 ± 20.91	51.38 ± 14.10	50.85 ± 14.22	44.84 ± 13.63	< 0.001
LDL cholesterol (mg/dL)	103.95 ± 31.09	118.55 ± 32.09	125.23 ± 33.62	116.02 ± 42.76	< 0.001
Triglycerides (mg/dL)	71.19 ± 14.91	109.59 ± 14.09	150.60 ± 23.77	286.05 ± 165.18	< 0.001
Dyslipidemia					
Yes	10 (5.3)	20 (10.6)	34 (17.9)	51 (27.0)	< 0.001
No	180 (94.7)	169 (89.4)	156 (82.1)	138 (73.0)	
Hypertension					
Yes	40 (21.1)	56 (29.6)	74 (38.9)	57 (30.2)	0.002
No	150 (78.9)	133 (70.4)	116 (61.1)	132 (69.8)	

All *p* < 0.001 between TyG index quartiles by analysis of variance post hoc analysis with the Bonferroni method

**Table 2:** Baseline characteristics of participants according to quartile of TG/HDL-C in pre-diabetes patients aged 35–70 years

Variables	TG/HDL-C				*P*-value
	Q1	Q2	Q3	Q4	
*N*	190	189	190	189	
Age (years)	52.63 ± 9.97	53.01 ± 8.78	53.22 ± 8.89	51.43 ± 9.16	0.236
Sex					
Male	70 (36.8)	73 (38.6)	68 (35.8)	98 (51.9)	0.004
Women	120 (63.2)	116 (61.4)	122 (64.2)	91 (48.1)	
Marital status					
Single	39 (20.5)	22 (11.6)	28 (14.7)	24 (12.7)	0.070
Married	151 (79.5)	167 (88.4)	162 (85.3)	165 (87.3)	
Education (year)	3.76 ± 3.82	3.47 ± 3.72	3.78 ± 3.74	4.07 ± 3.78	0.490
Socioeconomic status					
Low	77 (40.5)	82 (43.4)	68 (35.8)	83 (43.9)	0.488
Moderate	41 (21.6)	31 (16.4)	41 (21.6)	30 (15.9)	
High	72 (37.9)	76 (40.2)	81 (42.6)	76 (40.2)	
Body mass index (kg/m2)	25.05 ± 4.98	26.58 ± 5.03	28.41 ± 5.31	28.07 ± 4.44	< 0.001
Waist circumference (cm)	91.69 ± 12.21	96.02 ± 12.96	100.21 ± 11.82	100.01 ± 10.70	< 0.001
Hip circumference(cm)	98.19 ± 9.50	100.55 ± 9.98	103.43 ± 9.94	102.43 ± 8.41	< 0.001
Smoking					
Yes	38 (20.0)	47 (24.9)	44 (23.2)	44 (23.3)	0.719
No	152 (80.0)	142 (75.1)	146 (76.8)	145 (76.7)	
Glucose (mg/dL)	113.90 ± 26.97	114.00 ± 27.79	114.96 ± 25.23	119.04 ± 34.79	0.264
Total cholesterol (mg/dL)	177.07 ± 36.16	191.85 ± 36.76	206.20 ± 38.45	218.07 ± 50.45	< 0.001
HDL cholesterol (mg/dL)	66.98 ± 20.28	52.65 ± 12.40	46.18 ± 9.26	40.10 ± 8.46	< 0.001
LDL cholesterol (mg/dL)	108.36 ± 32.58	117.33 ± 34.12	122.80 ± 34.60	115.25 ± 40.78	0.001
Triglycerides (mg/dL)	75.12 ± 21.81	111.43 ± 26.68	147.73 ± 31.75	283.13 ± 166.17	< 0.001
Dyslipidemia					
Yes	19 (10.0)	23 (12.2)	29 (15.3)	44 (23.3)	0.002
No	171 (90.0)	166 (87.8)	161 (84.7)	145 (76.7)	
Hypertension					
Yes	42 (22.1)	63 (33.3)	64 (34.7)	56 (29.6)	0.034
No	148 (77.9)	126 (66.7)	124 (65.3)	133 (70.4)	

All *p* < 0.001 between TyG index quartiles by analysis of variance post hoc analysis with the Bonferroni method

Participants were stratified into four groups according to the quartiles of the TG/HDL-c and TyG indices. Age (*P* = 0.030), BMI (*P* < 0.001), WC (*P* < 0.001), HC (*P* < 0.001), TG (*P* < 0.001), total cholesterol (*P* < 0.001), FBS (*P* < 0.001), HDL-c (*P* < 0.001), LDL-c (*P* < 0.001), dyslipidemia (*P* < 0.001), and hypertension (*P* = 0.002) were statistically different between TyG quartiles. The results were similar for gender, BMI, WC, HC, total cholesterol, HDL-c, LDL-c, TG, dyslipidemia, and hypertension (*P* < 0.05) for TG/HDL-c quartiles (Tables 1 and 2).

**Fig. 2:**
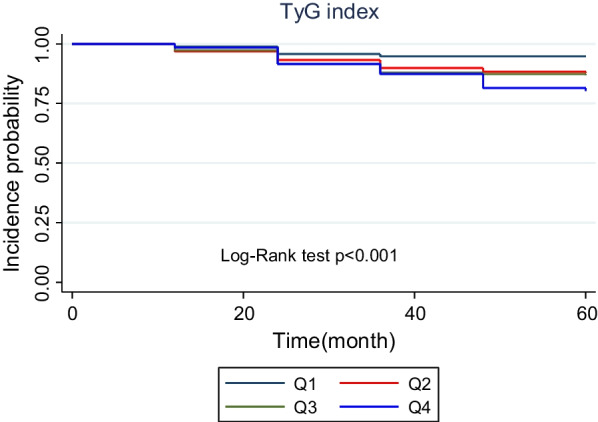
The cumulative incidence of type 2 diabetes by Kaplan–Meier analysis according to TyG index. The *p*-value was calculated with the log-rank test

**Fig. 3:**
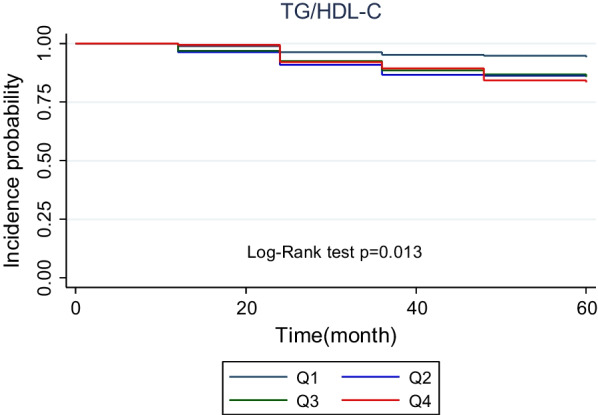
The cumulative incidence of type 2 diabetes by Kaplan–Meier analysis according to TG/HDL-C index. The *p*-value was calculated with the log-rank test

There were 95 incident cases of T2DM, with an overall incidence rate of 12.53%, during the 60-month follow-up. As shown in Table 3 in comparison with individuals in the lowest quartile of TyG and TG/HDL-c indices, participants in higher quartiles showed an increased cumulative incidence. Figures 2 and 3 depict the Kaplan–Meier curves for cumulative incidences of T2DM for TyG and TG/HDL-c indices quartiles. The highest TyG and TG/HDL-c indices quartile was associated with the highest probability of developing T2DM; these probabilities decreased sequentially for lower quartiles (*P* < 0.05) (Figs. 2 and 3).

**Table 3:** Risk of type 2 diabetes according to TyG index and TG/HDL-C quartile

Index	Events (*n*)	Population at risk	Cumulative incidence (95% CI)	Model 1, hazard ratio (95% CI)	Model 2, hazard ratio (95% CI)	Model 3, hazard ratio (95% CI)
*TyG index*						
Q1	10	190	5.26 (2.52–9.67)	1 (ref.)	1 (ref.)	1 (ref.)
Q2	23	189	12.16 (7.71–18.26)	2.37 (1.13–4.98) 0.022	2.40 1.14–5.05) 0.021	2.21 (1.01–4.80) 0.045
Q3	25	190	13.15 (8.52–19.42)	2.58 (1.24–5.37) 0.011	2.62 (1.25–5.46) 0.010	2.48 (1.11–5.53) 0.026
Q4	37	189	19.57 (13.78–26.98)	3.84 (1.91–7.72) < 0.001	3.84 (1.91–7.74) < 0.001	4.42 (1.75–11.21) 0.002
*TG/HDL-C*						
Q1	11	190	5.78 (2.89–10.35)	1 (ref.)	1 (ref.)	1 (ref.)
Q2	26	189	13.76 (8.99–20.16)	2.48 (1.22–5.02) 0.012	2.48 (1.22–5.03) 0.011	2.28 (1.11–4.68) 0.024
Q3	27	190	14.21 (9.36–20.68)	2.53 (1.25–5.11) 0.009	2.54 (1.26–5.13) 0.009	2.02 (0.97–4.17) 0.057
Q4	31	189	16.40 (11.14–23.28)	2.90 (1.45–5.77) 0.002	2.88 (1.44–5.76) 0.003	2.15 (1.04–4.47) 0.038

Model 1: crude, model 2: adjusted for age and sex

Model 3: adjusted for age, sex, smoking, marital status, socioeconomic status, body mass index, waist circumference, hip circumference, hypertension, total cholesterol, dyslipidemia,

Table 3 shows the results of multiple Cox proportional hazards regression analyses. In model 1 and model 2 (adjust for age and sex), a higher TyG and TG/HDL-C had a significant association with incident T2DM in pre-diabetic patients. TyG quartiles compared with Q1, HRs (95%CI, *p*-value) for the incidence of T2DM in Q2, Q3, and Q4 were 2.21 (1.01–4.80, *P* = 0.045), 0.48 (1.11–5.53, *P* = 0.026), and 4.42 (1.75–11.21, *P* = 0.002), respectively, after adjusting for age, sex, smoking, marital status, socioeconomic status, BMI, WC, HC, hypertension, dyslipidemia, FBS, TG, and HDL-C (Model 3). Also, with an increscent TG/HDL-c quartile compared with the first quartile, the risk of T2DM increases in Q2 (HR: 2.28, 95% CI: 1.11–4.68, *P* = 0.024) and Q4 (HR: 2.15, 95% CI: 1.04–4.47, *P* = 0.038), respectively, in model 3.

## Discussion

The results of this prospective cohort study showed that TyG and TG/HDL-c indices are independently and positively related to the risk of T2DM development in pre-diabetic patients. Also, an increasing trend of T2DM risk was seen with increasing in TyG and TG/HDL-c indices score after adjustment of the confounding variables of age and sex.

Several previous prospective studies showed that increasing TyG and TG/HDL-c indices increase the risk of T2DM [26–28]. A community-based cohort study in China with 687 participants and a 15-year follow-up period showed that an increment in the TG/HDL-c increases the risk of T2DM (OR = 1.34, *P* = 0.1) [8]. The TyG and TG/HDL-c indices are closely related to insulin resistance, and these indices may be a proxy for insulin resistance to identify individuals with a high risk of T2DM [29]. Also, alternative markers of insulin resistance have been introduced to predict the risk of T2DM such as lipoprotein particles’ concentration and size, glucose, and insulin levels. However, recent studies suggest that TyG and TG/HDL-c indices may be better predictors of insulin resistance and cardiovascular disease risk than single indicators [9, 30].

High levels of TG and low levels of HDL-c are two key metabolic abnormalities [30]. Therefore, high TG/HDL-c may indicate individuals have a metabolic disorder with higher BMI and higher levels of FBS, TG, LDL-c, and very low-density lipoprotein (VLDL-c) and lower levels of HDL-c. Thus, this evidence supports that the above-mentioned index may be a good predictor of T2DM risk [31]. The mechanisms by which the high TG/HDL-c causes T2DM are complicated and unclear. Investigations showed that a higher level of triglycerides can increase the plasma free fatty acid levels, which increases the level of triglycerides and decreases the level of HDL-c in the liver, consequently accelerating insulin resistance and contributing to the development of T2DM [32]. A series of abnormalities related to insulin resistance such as B-cell dysfunction, increased blood glucose, impaired insulin secretion, and B-cell apoptosis can be caused by excessive triglyceride and low HDL-c levels. In addition, increased levels of nitric oxide and ceramide due to increased levels of TG can induce B-cell apoptosis. Accumulation of cholesterol in B cells as a result of low HDL-c can cause cytotoxicity [16]. In addition, low HDL-c levels can directly mediate glucose uptake and thereby lead to T2DM [11].

Central obesity, insulin resistance, dyslipidemia, and hypertension increase the risk of cardiovascular diseases and T2DM. Triglyceride and HDL-c are important risk factors for cardiovascular diseases [33]. Babic et al. [34] showed that this ratio may be a surrogate metabolic variable that predicts the development of T2DM among individuals. In addition, to describe diabetes through insulin resistance, T2DM is also characterized by a decrease in insulin secretion. Therefore, pancreatic B-cell dysfunction can also be one of the possible explanations for the relationship between the TG/HDL-c index and T2DM [16].

TyG index, which is a combination of TG and FBS, is a simple, effective, reproducible, and reliable alternative for insulin resistance [35]. Previous studies showed that fasting blood glucose and TG reflect insulin resistance in the liver and fat cells, respectively. Therefore, the increase in the TyG index over time reflects insulin resistance in both liver and fat cells [36].

The present study faces some limitations. One of the limitations of the study is the identification of T2DM through fasting blood glucose instead of the 2-h oral glucose tolerance test or measuring the level of HbA1c, which may underestimate the incidence of T2DM. It should be noted that the information was obtained from the Iranian population who are at risk of T2DM and the generalizability of the results is limited due to similar genetic characteristics and lifestyle. Finally, a healthy diet can affect fat homeostasis and glucose metabolism, which the dietary data were not evaluated in our study. Studies are needed to determine how a diet affects triglycerides and T2DM risk.

## Conclusion

The results from this study showed that a high TyG and TG/HDL-c index is associated with an increased incidence of diabetes in pre-diabetes patients. Therefore, it is necessary to lower the level of TyG and TG/HDL-c through preventive measures such as lifestyle management for middle-aged people.
